# Getting a *grippe* on severity: a retrospective comparison of influenza-related hospitalizations and deaths captured in reportable disease and administrative data sources in Ontario, Canada

**DOI:** 10.1186/s12889-019-6924-9

**Published:** 2019-05-14

**Authors:** J. Leigh Hobbs, Michael Whelan, Anne-Luise Winter, Michelle Murti, Karin Hohenadel

**Affiliations:** 1 Public Health Ontario, 480 University Avenue, Suite 300, Toronto, Ontario M5G 1V2 Canada; 2 Dalla Lana School of Public Health, University of Toronto, 155 College Street, Toronto, Ontario M5T 3M7 Canada

**Keywords:** Influenza, Reportable disease data, Administrative data, Hospital data, Discharge data, Surveillance, Indicators of severity, Retrospective

## Abstract

**Background:**

Since 2009, in Ontario, reportable disease surveillance data has been used for timely in-season estimates of influenza severity (i.e., hospitalizations and deaths). Due to changes in reporting requirements influenza reporting no longer captures these indicators of severity, necessitating exploration of other potential sources of data. The purpose of this study was to complete a retrospective analysis to assess the comparability of influenza-related hospitalizations and deaths captured in the Ontario reportable disease information system to those captured in Ontario’s hospital-based discharge database.

**Methods:**

Hospitalizations and deaths of laboratory-confirmed influenza cases reported during the 2010–11 to 2013–14 influenza seasons were analyzed. Information on hospitalizations and deaths for laboratory-confirmed influenza cases were obtained from two databases; the integrated Public Health Information System, which is the provincial reportable disease database, and the Discharge Abstract Database, which contains information on all in-patient hospital visits using the International Classification of Diseases, 10th Revision, Canada (ICD-10-CA) coding standards. Analyses were completed using the ICD-10 J09 and J10 diagnosis codes as an indicator for laboratory-confirmed influenza, and a secondary analysis included the physician-diagnosed influenza J11 diagnosis code.

**Results:**

For each season, reported hospitalizations for laboratory-confirmed influenza cases in the reportable disease data were higher compared to hospitalizations with J09 and J10 diagnoses codes, but lower when J11 codes were included. The number of deaths was higher in the reportable disease data, whether or not J11 codes were included. For all four seasons, the weekly trends in the number of hospitalizations and deaths were similar for the reportable disease and hospital data (with and without J11), with seasonal peaks occurring during the same week or within 1 week of each other.

**Conclusion:**

In our retrospective analyses we found that hospital data provided a reliable estimate of the trends of influenza-related hospitalizations and deaths compared to the reportable disease data for the 2010–11 to 2013–14 influenza seasons in Ontario, but may under-estimate the total seasonal number of deaths. Hospital data could be used for retrospective end-of-season assessments of severity, but due to delays in data availability are unlikely to be timely estimates of severity during in-season surveillance.

## Background

Infection with the influenza virus can cause significant morbidity and mortality, especially in high-risk populations [1]. In Canada, it has been estimated that influenza causes approximately 12,200 hospitalizations and 3500 deaths each year [1]. Accurately assessing seasonal influenza-related morbidity and mortality is challenging since influenza-related hospitalizations are under-reported because symptoms and complications (e.g., cardiac complications) are non-specific. Additionally, individuals presenting for care with influenza may be diagnosed with exacerbations of a chronic disease (e.g., heart failure, chronic obstructive pulmonary disease) rather than acute influenza infection. This may result in reduced laboratory testing of patients hospitalized with influenza, leading to less detection and reporting of the virus [2]. Further, testing biases are observed in less severe influenza seasons, which also impacts detection and reporting [3].

Reportable disease surveillance systems are critical in providing timely estimates of severity during the current season in order to inform influenza control measures, as well as allowing for comparison of seasonal severity as an early indicator of unusual influenza activity [4, 5]. The World Health Organization recommends that all information required for pandemic influenza surveillance be routinely collected for seasonal surveillance as the resources and processes to collect this information cannot be immediately scaled up [5]. Since 2009 in Ontario, reportable disease surveillance data for influenza, which is reliant on public health follow-up of laboratory-confirmed influenza cases, has been used for timely in-season provincial estimates of influenza-related hospitalizations and deaths. Surveillance data are further used to inform influenza case and outbreak management, such as the early use of antiviral drugs, to promote control measures, including influenza vaccination, as well as to allow for comparison of seasonal severity as an early indicator of unusual influenza activity.

Changes in reporting requirements and public health follow-up of laboratory-confirmed influenza cases in Ontario occurred between 2014 and 2017 and, on January 1, 2018, the *Health Protection and Promotion Act* was updated such that public health follow-up of individuals with seasonal laboratory-confirmed influenza was no longer required [6]. Public health was no longer required to obtain information on outcomes such as hospitalization and death for reported cases of seasonal influenza. As a result of these changes, it is no longer possible to obtain timely estimates of in-season severity (such as hospitalizations and deaths) for influenza from reportable disease surveillance data in Ontario. Therefore, alternate data sources are needed.

Administrative data, such as hospital-based databases, are another potential source to assess influenza-related hospitalizations and deaths. Published studies have described administrative databases as a good source of population-based disease information for retrospective estimates of influenza severity and burden of disease [4, 7, 8]. While administrative databases are unlikely to be sufficiently timely to inform in-season influenza estimates of severity, they could provide an alternative source of information for retrospective end-of-season assessments of influenza trends and severity [9, 10].

The purpose of our study was to complete retrospective analyses to assess the end-of-season comparability of influenza-related hospitalizations and deaths captured in the Ontario reportable disease information system to those captured in a hospital-based database which includes all hospitals (over 220) in the province of Ontario.

## Methods

### Reportable disease data (integrated public health information system)

Information on hospitalizations and deaths for incident reports of influenza (meeting the provincial case definition for laboratory-confirmed cases, as described below) with episode dates from September 1, 2010 to August 31, 2014 were obtained through the Ontario Ministry of Health and Long-Term Care’s (MOHLTC) integrated Public Health Information System (iPHIS), the provincial reportable disease database in Ontario. iPHIS data can be requested through the MOHLTC. Episode date is a calculated field for all cases in the reportable disease database and cannot be blank. The episode date is calculated using the first available of symptom onset date, or if missing the laboratory specimen collection date, or if missing date reported to public health. An influenza season was defined as September to August of the following year.

Public health units in Ontario were required, under provincial legislation, to report and enter information on cases of influenza reported to them by health care providers or laboratories into iPHIS. As a result of an increased need for information about influenza following the 2009 H1N1 influenza A pandemic, public health units were also requested to follow-up with all laboratory-confirmed cases of influenza in order to obtain information on case demographics, symptom onset, and outcomes (e.g., hospitalization and death). All case information was required to be entered in iPHIS within 30 days of follow-up completion; however, case information in iPHIS is available in real time (i.e., case information is available as it entered).

Post-pandemic case follow-up for hospitalizations and deaths for reported cases of influenza was most complete and consistent for the 2010–11 to 2013–14 seasons in Ontario. Reporting requirements changed starting in December 2014 when public health units were only required to follow-up every fifth case of influenza to ascertain hospitalization or death outcomes, and in 2018, public health units were no longer required to follow-up with any seasonal influenza cases.

A hospitalization was defined as having at least one hospital admission date associated with a laboratory-confirmed case, and death was defined as any fatal outcome reported at the time of follow-up in a laboratory-confirmed case. As per standard reporting practices in Canada, this included cases where the cause of death was not reported, reported as not determined, or was reported as not attributed to influenza [11]. Influenza cases in iPHIS were entered according to the surveillance case definition in use at the time of case reporting, the most recent of which has been in effect since December 2014 [12]. Cases of influenza may be confirmed by a variety of laboratory tests (e.g. nucleic acid amplification test for influenza virus RNA, influenza virus culture, etc.) as described in the provincial case definition [12]. There were no changes to the confirmed case definition during the 2010–11 to 2013–14 seasons included in the analysis.

### Hospital administration data (discharge abstract database)

Information on influenza-related hospital admissions and deaths were obtained from the Canadian Institute for Health Information’s (CIHI) Discharge Abstract Database (DAD), which is populated with data directly from acute care facilities across Canada or their respective health authority or ministry. Reporting to the DAD by hospitals is required in Ontario [13]. Records are coded at patient discharge using the International Statistical Classification of Diseases and Related Health Problems, 10th Revision, Canada (ICD-10-CA) coding standards [14]. In Ontario, DAD data are made available to provincial and local public health agencies through a data retrieval platform, IntelliHealth Ontario, and updated data are available four times per year, following a quarterly refresh. Data available at each refresh would be roughly 4 to 6 months old depending on when a patient was discharged. IntelliHealth Ontario data can be requested through the MOHLTC.

Influenza-related hospitalizations with available admission dates (intended to capture symptom onset dates) from September 1, 2010 to August 31, 2014 were obtained from DAD. An influenza-related hospitalization was defined as any admission with one of the following diagnoses codes: the ICD-10 J09 and J10 codes indicating virus identified (intended to capture laboratory-confirmed influenza) or the J11 code indicating virus not identified (intended to capture physician-diagnosed influenza) [2, 15]. As influenza-related admissions may be the result of a complication (e.g., pneumonia) coded as the primary diagnoses, all diagnoses (i.e., the most responsible diagnoses and up to 25 additional diagnoses, such as pre-admission comorbidity, etc.) were included [2, 15, 16]. Other studies have used varying methods to exclude hospital transfers and re-admissions from discharge data for influenza and other reportable diseases [2, 4]. In order to exclude hospital transfers or re-admissions associated with the same infection, we only included the first admission within a 30-day interval. A death was defined as any admission with a discharge status reported as fatal.

### Analyses

The analyses included four historical influenza seasons from 2010 to 11 to 2013–2014. The number of hospitalizations and deaths were summarized by influenza season and by epidemiological surveillance week for both the reportable disease (using episode date) and hospital data (using admission date). Analysis for the hospital data was completed using the ICD-10 J09 and J10 diagnosis codes, and a secondary analysis was done which also included the J11 diagnosis code. Descriptive analyses were performed using SAS 9.3 (SAS Institute Inc., Cary, NC, USA) and figures were constructed using Microsoft Excel (Microsoft Corporation, version 2010, Redmond, WA, USA). Linkage between the hospital and reportable disease data was not performed for this analysis. This study did not require research ethics committee approval as the activities described in were considered public health practice, not research, and were conducted in fulfillment of Public Health Ontario’s legislated mandate as outlined in *the Ontario Agency for Health Protection and Promotion Act, SO 2007, c 10*.

## Results

### Reportable disease data: episode date

For the reportable disease data, an onset date was available and used to determine the episode date for the majority of laboratory-confirmed influenza cases (84%), followed by a specimen collection date for 14% of cases and a reported date for 2% of cases. For laboratory-confirmed influenza cases where hospitalization and/or death were reported, an onset date was available for 94 and 89% of cases, respectively.

### Trends over time: hospitalizations

For each season, the total number of hospitalizations for laboratory-confirmed influenza cases captured in the reportable disease data was higher compared to those coded as laboratory-confirmed influenza hospitalizations (J09 and J10 diagnosis codes) in the hospital data (Table 1). However, when hospitalizations with a J11 code indicating physician-diagnosed influenza were included, the total number of hospitalizations captured in the hospital data was higher compared to laboratory-confirmed hospitalizations captured in the reportable disease data. This trend was consistent for each of the four seasons.

**Table 1:** Total number of laboratory-confirmed influenza cases, hospitalizations and deaths captured in reportable disease and hospital data, Ontario, 2010–11 to 2013–14 influenza seasons

	Season				
	2010–11	2011–12	2012–13	2013–14	Totals
Reportable disease data					
Influenza cases	6048	3946	9782	9958	29,734
Hospitalizations	2380	1243	3699	3738	11,060
Deaths	202	79	292	261	834
Hospital data					
Hospitalizations (J09, J10)	1916	874	3099	2943	8832
Hospitalizations (J09, J10, J11)	2778	1282	4079	3770	11,909
Deaths (J09, J10)	126	54	200	193	573
Deaths (J09, J10, J11)	185	61	253	220	719

For reportable disease data, hospitalizations and deaths are counted using the episode date (the first of onset date, laboratory specimen collection date, or date reported to public health)

For hospital data, hospitalizations and deaths are counted using the admission date (as an indicator for date of symptom onset)

**Fig. 1:**
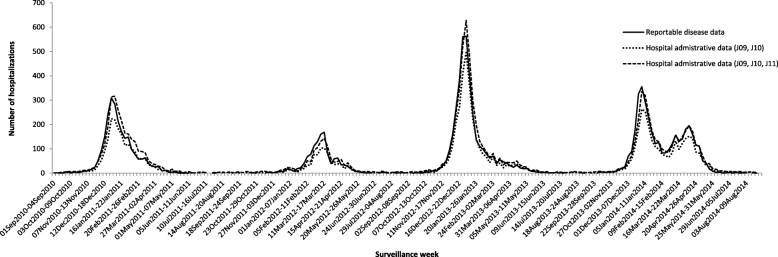
Number of influenza-related hospitalizations captured in reportable disease and hospital discharge data by epidemiological surveillance week, Ontario, 2010–11 to 2013–14 influenza seasons

For all four seasons, the weekly trends in the number of hospitalizations were similar across the reportable disease and hospital data, whether or not the J11 code indicating physician-diagnosed influenza was included (Fig. 1). For the 2010–11 season, a similar increasing and decreasing trend with a peak at the end of December and/or beginning of January was observed for the reportable disease and hospital data (both with and without the J11 diagnoses code). The peak number of hospitalizations was earlier (week of December 26, 2010 to January 1, 2011) for both the reportable disease data and hospital data for laboratory-confirmed influenza (J09 and J10 diagnoses codes) compared to the hospital data that included physician-diagnosed influenza (J09, J10 and J11 diagnosis codes), which occurred the following week (January 2 to 8, 2011). Results were similar for the 2011–12 season. The peaks for the laboratory-confirmed reportable disease data and hospital data that included the J11 physician-diagnosed influenza (J09, J10 and J11 diagnosis codes) were observed the week of March 11 to 17, 2012, and the peak for the laboratory-confirmed hospital data (J09 and J10 diagnoses codes) began 1 week earlier spanning the March 4 to 17, 2018 period.

For the 2012–13 and 2013–14 seasons the peak was observed during the same week (December 30, 2012 to January 5, 2013 and December 29, 2013 to January 4, 2014, respectively) for the reportable disease and hospital data (with and without the J11 diagnosis code). In the 2013–14 season, during a second wave of activity from March to April due to influenza B, an additional peak occurred in April (April 6 and 12, 2014) in the reportable disease and hospital data (with and without J11). When all four influenza seasons are compared, the highest peak number of hospitalizations was observed in the 2012–13 season. The peak was highest in this season for the reportable disease and hospital data (with and without J11) as a result of a severe H3N2 influenza A season.

### Trends over time: deaths

**Fig. 2:**
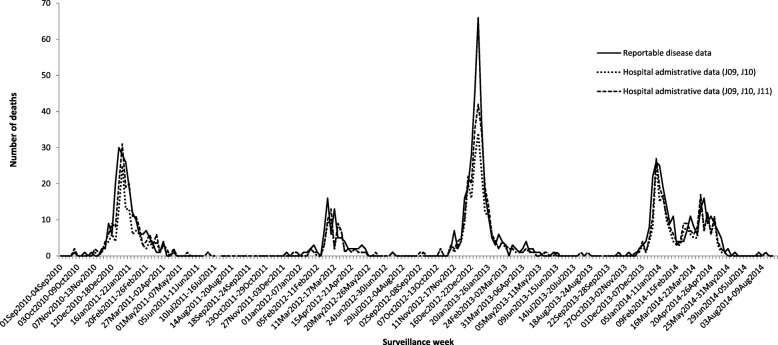
Number of influenza-related deaths captured in reportable disease and hospital discharge data by epidemiological surveillance week, Ontario, 2010–11 to 2013–14 influenza seasons

For deaths reported in influenza cases, the total number of deaths for each season was higher in the laboratory-confirmed reportable disease data compared to the number of deaths captured in the hospital data, whether or not the J11 code indicating physician-diagnosed influenza was included (Table 1). The weekly trends in influenza-related deaths were also similar for each season for both the reportable disease and hospital data (Fig. 2). For the 2010–11 and 2011–12 seasons, the peak number of deaths for the laboratory-confirmed reportable disease data was 1 week earlier (December 26, 2010 to January 1, 2011 and February 28 to March 3, 2012, respectively), compared to the peak for the hospital data (January 2 to 8, 2011 and March 4 to 10, 2012, respectively). This trend was consistent whether or not the J11 code indicating physician-diagnosed influenza was included.

For the 2012–13 and 2013–14 seasons, the peak number of influenza-related deaths occurred in the same week (December 30, 2012 to January 5, 2013 and December 29, 2013 to January 4, 2014, respectively) for the reportable disease and hospital data (both with and without J11). In the 2013–14 season, during a second wave of activity due to influenza B, a peak was observed later in the laboratory-confirmed reportable disease data (April 6 to 12, 2014) compared to the hospital data (both with and without J11). The second wave of activity for the hospital data occurred earlier during the week of March 30 to April 5, 2014.

## Discussion

In our retrospective analyses we found that hospital administrative data (for influenza ICD 10 codes J09-J11) provided a reliable estimate of trends of influenza-related hospitalizations and deaths, as well as the total seasonal numbers of hospitalizations, compared to the reportable disease data for the 2010–11 to 2013–14 influenza seasons in Ontario. However, the total seasonal numbers of deaths were lower in the hospital data compared to the reportable disease data.

Our results showed that for influenza, reportable disease and hospital data followed the same temporal trends with only minor variations. The peak number of hospitalizations and deaths were observed during the same week or within 1 week of each other and were not consistently earlier in one data source. This is a similar finding to another study that compared temporal trends for ICD-10 codes for severe acute respiratory infection against influenza positive samples collected from an outpatient sentinel surveillance system [2, 3]. Further, our results included seasons of varying severity as well as different dominant influenza subtypes, and consistently showed that the trends in influenza hospitalization and deaths were similar in reportable disease and hospital data. For the 2012–13 season, which was the most severe of the four seasons, the highest numbers of hospitalizations and deaths were observed in both data sources. Ontario surveillance reports for the 2012–13 season similarly indicated high influenza activity with H3N2 influenza A as the dominant subtype which may have contributed to a higher number of hospitalizations and deaths compared to other seasons, whereas H1N1 was the dominant subtype in the other seasons included in our analysis [17]. The consistency in the total seasonal number of influenza-related hospitalizations as well as the weekly trends (including seasonal peaks) across the two data sources suggests hospital data is a reasonable alternative to reportable disease data for retrospective end-of-season assessments of trends in weekly influenza activity and hospitalizations.

However, hospital data may provide a less accurate estimate of the total seasonal number of deaths for influenza compared to reportable disease data. For all four seasons, the number of deaths captured in hospital data (whether or not the J11 physician-diagnosed influenza code was included) was less than the number of deaths in the reportable disease data. This may indicate a number of deaths occurred in long-term care homes, or in community settings (such as following hospital discharge) thus were not captured in hospital data. This finding could also be reflective of the non-specific nature of death reporting in the reportable disease database, where any fatal outcome for a laboratory-confirmed case of influenza (whether or not the death was attributed to the influenza infection) is reported as a death. Since influenza infection may not be recognized as a cause or contributing cause of death for hospitalized patients, reportable disease data may be more reflective of the number of deaths associated with influenza infection compared to hospital data. Further, in our study, in order to exclude hospital transfers and re-admissions associated with the same infection, only the first admissions within a 30-day interval were included. As a result, deaths that occurred following re-admission or transfer to another hospital would have been excluded.

Studies elsewhere that completed record level linkages of influenza laboratory and hospital data found mixed results for the sensitivity of ICD discharge codes. Some record level linkage studies found high accuracy for ICD discharge codes for laboratory-confirmed influenza (i.e., high sensitivity), while other studies reported lower sensitivity of ICD discharge codes [2, 15, 16, 18]. One study concluded hospital data cannot accurately determine the incidence of influenza-related hospitalizations and deaths in a population, but that it could be a useful indicator for assessing trends over time [2]. A study linking reportable disease and hospital data found the sensitivity of ICD-9 codes (which are similar to ICD-10 codes) varied by disease [4]. Sensitivity depended on the complexity of the reportable disease case definition, as well as the usefulness of clinical signs and symptoms alone in making the diagnosis. Discharge diagnoses were most useful in correctly identifying cases of reportable diseases with simple case definitions and non-specific clinical signs and symptoms, thus requiring laboratory confirmation prior to diagnoses [4]. Influenza fits this description in terms of having non-specific clinical presentation requiring laboratory confirmation; however the study did not include influenza.

Similar to studies elsewhere, the use of hospital data for timely in-season influenza surveillance in Ontario would be limited by the delay in data availability [3]. Reportable disease data is collected for surveillance purposes and is available in real time, while hospital discharge data is collected for administrative and billing purposes and is coded upon patient discharge, which may be long after admission (i.e., when influenza infection is deemed to have occurred). Data are then made available following a quarterly refresh, and are at least 4 to 6 months old depending on when a patient was discharged and a patients’ length of stay. As a result, hospital data would likely not provide timely weekly estimates of in-season severity in Ontario.

Underreporting of influenza due to ill individuals not seeking health care as well as variation in clinician testing practices with seasonal severity are important considerations in any analysis of laboratory-confirmed influenza cases; however, this would likely impact both reportable disease and hospital data. The sensitivity of the testing method used to identify laboratory-confirmed cases is also an important consideration. More sensitive molecular tests, which result in improved laboratory detection, are not always economically feasible or available, thus their use likely varies across types of hospitals. This could impact both reportable disease and/or hospital data depending on where more sensitive tests are being used [2].

Mandatory reporting and follow-up of laboratory-confirmed influenza cases in a reportable disease database likely ensures complete ascertainment of cases and case details that are available at the time of follow-up, including deaths that occurred in a hospital or non-hospital setting. However, case follow-up is done at a cross-section in time. Hospitalizations and deaths that occurred after case follow-up was complete would not be captured. Muscatello noted a lag in the time frame from specimen collection to the date of death requiring follow-up for an extended period of time in order to report this outcome in a public health surveillance system [2].

In theory, hospital data should capture all influenza-related hospitalizations, as well as deaths occurring in the hospital. However, capturing influenza-related hospitalizations and deaths is dependent on appropriate ICD-10 coding, and as discussed above, studies have found varying sensitivities for ICD-10 coding in detecting laboratory-confirmed influenza [2, 15, 16, 18]. ICD coding sensitivity depends on a number of factors, such as the type of discharge diagnoses included (e.g., primary versus all diagnoses) [15, 16]. Further, hospitalized patients with influenza-related complications, even with laboratory-confirmed influenza infection, may only have non-influenza discharge codes, such as pneumonia [2, 15, 18]. Discharge diagnoses may therefore not capture influenza infection for patients with severe illness. Short lengths of hospital stay for patients may also impact coding. Confirmatory laboratory test results may not be available at the time of discharge affecting the ICD-10 coding entered on discharge from hospital. The result would be that these patients with short lengths of stay would not be captured in hospital data that we analyzed [3, 18]. Further, confirmatory testing done in the community prior to admission may not be captured in hospital data [2]. Population characteristics may also influence appropriate ICD coding for laboratory-confirmed influenza hospitalizations. For example, accuracy of coding was improved for paediatric and older patient populations [2, 18]. Sensitivity of coding is also known to vary with residing in remote areas as compared to larger cities [2, 16]. Misclassification and coding errors were also identified. For example, influenza virus may be coded as *Haemophilus influenza*, a bacterial infection, and vice versa [2]. Assigning ICD codes is based on coding standards, not established case definitions with approved or validated laboratory tests required for confirmation. Thus, a discharge diagnosis may not meet the criteria for a confirmed case according to the surveillance case definitions [4]. For example, a point of care test not done in a laboratory could be reported as confirmed influenza in hospital data, but may not meet the surveillance case definition. Assessing the impacts of these ICD-10 coding considerations on our analysis would require record-level linkage of cases, which was out of scope for our study.

Despite the limitations noted above regarding accurately capturing influenza-related hospitalizations and deaths in hospital data, they do not appear to have a substantial impact on the overall number of influenza-related hospitalizations captured in hospital data for the four seasons described, although may have a larger impact on the total number of deaths. Additionally, the limitations do not appear to impact the accuracy of the weekly trends in influenza-related hospitalizations and deaths.

## Conclusions

In our retrospective analysis we found that hospital data provided a reliable estimate of the weekly trends of influenza-related severity indicators (i.e., hospitalizations and deaths), as well as the total seasonal numbers of hospitalizations, but under-estimated the total seasonal number of deaths, compared to the reportable disease data for the 2010–11 to 2013–14 influenza seasons in Ontario. Hospital data could therefore be used for retrospective end-of-season assessments of influenza trends and severity (but may provide an under-estimate of the total seasonal deaths) and could be used to inform program planning and communication for future seasons. However, due to delays in data availability, hospital data in Ontario could likely not be used for timely weekly in-season estimates of severity in order to promote control measures or as an early indicator of unusual influenza activity. Further, to the authors’ knowledge, a comparison of reportable disease and hospital data has not previously been completed for influenza.

Future studies in Ontario could complete a similar analysis of hospital data for the 2014–15 to 2016–17 influenza seasons, when public health follow-up was not complete for laboratory-confirmed influenza, in order to retrospectively compare seasonal trends and severity in years where follow-up was incomplete. Additionally, a chart audit to determine the accuracy of ICD-10 discharge coding for influenza and a record level linkage of reportable disease and hospital administrative data to laboratory data could help inform the accuracy of ICD 10 discharge coding for measuring influenza-related hospitalizations and deaths and to what extent the limitations of hospital data described above are applicable in Ontario.

We did not assess the timeliness of reporting, thus did not evaluate the utility of discharge data for measuring severity for surveillance during the influenza season. Further analysis could also assess the extent of the differences in timeliness of reporting of influenza-related hospitalizations and deaths in reportable disease data compared to hospital administrative data. An analysis of reporting delays in reportable disease data and length of stay and re-admissions in hospital administrative data could offer insights into the practicality of using hospital discharge data for timely in-season surveillance. Finally, future similar studies from other jurisdictions would provide insight on the generalizability of these results to settings outside of the Ontario and Canadian healthcare system context.

## Abbreviations

CIHI: Canadian Institute for Health Information
DAD: Discharge Abstract Database
ICD: International Statistical Classification of Diseases and Related Health Problems
iPHIS: Integrated Public Health Information System
MOHLTC: Ministry of Health and Long-Term Care
